# From Microbiome to Inflammation: The Key Drivers of Cervical Cancer

**DOI:** 10.3389/fmicb.2021.767931

**Published:** 2021-11-15

**Authors:** Zi-Wei Zhou, Hui-Zhi Long, Yan Cheng, Hong-Yu Luo, Dan-Dan Wen, Li-Chen Gao

**Affiliations:** ^1^Department of Pharmacy, Cancer Institute, Phase I Clinical Trial Centre, The Affiliated Changsha Central Hospital, Hengyang Medical School, University of South China, Hengyang, China; ^2^Hunan Provincial Key Laboratory of Tumor Microenvironment Responsive Drug Research, Hengyang, China

**Keywords:** microbiome, inflammation, cervical cancer, probiotic, immunotherapy

## Abstract

Cervical cancer is the third leading cause of cancer-related death worldwide. Microbes and hosts form a mutually beneficial symbiosis relationship, and various parts of the host body are microbial habitats. Microbes can trigger inflammation in certain parts of the host body, contributing to cervical cancer development. This article reviews the relationship between cervicovaginal microbes, inflammation and cervical cancer, and discusses the effect of some key cervical microbes on cervical cancer. Finally, probiotic therapy and immunotherapy are summarized.

## Introduction

Cervical cancer is one of the most significant malignancies in females, and the third leading cause of cancer-related deaths worldwide. It has been estimated that there are about 530,000 new cases and 275,000 deaths worldwide each year (Olusola et al., 2019). Cervical cancer is the most common cancer in Eastern Africa (Ferlay et al., 2019). The cervix surface comprises two kinds of epithelial cells layers: the outer squamous cells and columnar glandular cells along the inner canal. The junction of columnar cells and squamous cells is termed the squamocolumnar junction. This junction is prone to precancerous lesions and canceration. In the early stages, cervical cancer is often unnoticed due to the inconspicuous symptoms. However, many common symptoms such as vaginal bleeding, abnormal vaginal discharge, and dyspareunia occur when cervical cancer advances to the terminal stage (Reed et al., 2021). Although the development of cervical cancer could be prevented by routine screening and other treatment approaches, mortality rates do not decrease significantly. It is urgently needed to explore standard treatments for cervical cancer.

Inflammation is a kind of defense mechanism to various stimuli. Tissue damage and various contributing factors can trigger inflammation. When the host body shows inflammatory signs, it results in the following phenomenon: elevated cellular metabolism, vessel wall dilatation, the release of soluble mediators and increased blood flow (Ferrero-Miliani et al., 2007). The inflammation period is classified into an acute period and a chronic period. Immune cells migrate to the injury site to initiate inflammation by regulating soluble mediators in the acute phase. Persistent inflammation contributes to the chronic period. The salient feature of chronic inflammation could be explained by lymphocytic infiltration. Antibodies or cytokines are secreted by T and B lymphocytes, which are involved in tissue damage and inflammatory cell recruitment. Chronic inflammation can lead to atherosclerosis, diabetes, aging, autoimmune diseases, and even cancers (Gabay and Kushner, 1999; Castellon and Bogdanova, 2016; Germolec et al., 2018).

The microflora is a collection of living microbes that live in biological organs. There are various microbes on the body’s surface, and the human body is a habitat of trillions of microbes (Adak and Khan, 2019). Complementing microbiome with humans is established a complex mutualistic host-microbial relationship. The human body provides a suitable living environment for microbes. The microbiome plays a crucial role in the development and normal function of the body, including modulating the immune system, absorbing nutrients and protecting the body (Van de Wiele et al., 2016; Jenmalm, 2017).

With the advent of research, the relationship between the microbiome and inflammation has become increasingly apparent. Moreover, the role of microbiome and inflammation in the occurrence and development of cancer has also been reported by numerous research studies. We hypothesized that when the homeostasis of microorganisms is compromised, the microorganisms themselves or their secretions will cause a series of immune responses in the organism. Persistent inflammation can result in chronic inflammation, which is one of the inducing factors of the tumor. In the presence of chronic inflammation, the organism’s susceptibility increases, making the cells prone to cancer. Most studies in cervical cancer have a focus on the relationship between microbiota and cervical cancer, yet inflammation may also be a key driver of cervical cancer. To determine the link between microbiota inflammation and cervical cancer, this review examines how the microbiota profiles, the protective effects of *Lactobacillus* and the association of microbiota disturbances under inflammation and human papillomavirus (HPV). We also explained the carcinogenic role of the typical cervical microbiome. Finally, probiotic therapy and immunotherapy are discussed.

## The Vaginal Microbial Environment

As in the gastrointestinal tract, the female reproductive tract is a habitat for microbes. Most microbes coexist with the body, affecting human health and disease (Foster et al., 2017). Researchers have analyzed the distribution of vaginal microbiome in women of reproductive age by 16S rRNA gene sequencing, and hierarchical taxonomic clustering, and they conclude that the vaginal microbiome profile of each woman could be classified into six community state types (CSTs) (Mitra et al., 2016; Brotman et al., 2018). CST-I, II, III, and V are dominated by *Lactobacillus crispatus*, *Lactobacillus gasseri*, *Lactobacillus iners*, and *Lactobacillus jensenii*, respectively. In contrast, CST-IV, has a low relative abundance of *Lactobacillus* and high relative abundance of facultative anaerobes, and could be divided into CST IV-A as well as CST IV-B. *Streptococcus* and *Prevotella* characterize CST IV-A, and *Atopobium* accounted for a higher proportion in CST IV-B. Importantly, anaerobic bacteria of CST-IV are commonly associated with bacterial vaginosis (BV). In general, healthy women have one or more *Lactobacillus* in the vagina, with a small diversity of bacteria (Laniewski et al., 2020).

### The Vaginal Microbial Environment of a Healthy Person

**FIGURE 1:**
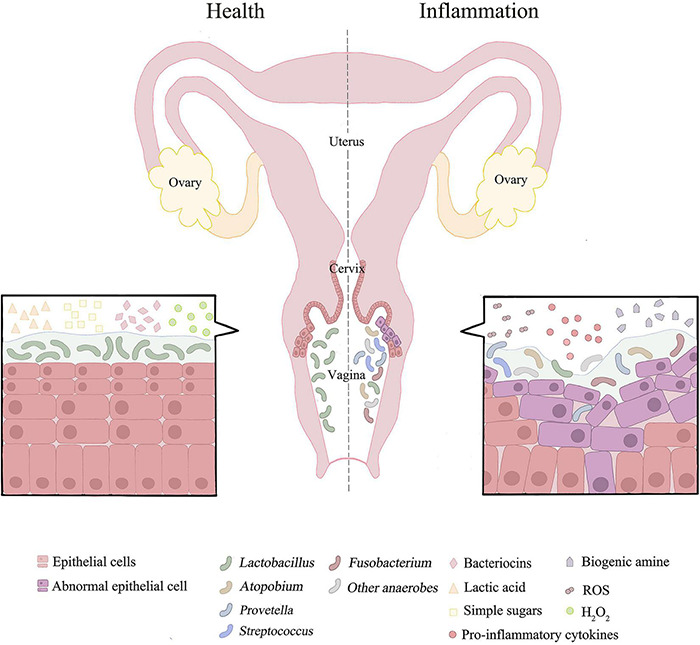
The distribution of vaginal microorganisms in a healthy or inflammatory state. The vaginal microbial environment of healthy people is usually dominated by *lactobacillus* with high abundance. *Lactobacillus* depends on glycogen products for growth. Lactic acid, bacteriocins and H_2_O_2_ produced by *Lactobacillus* may inhibit the growth and development of pathogenic bacteria. When female reproductive tract is in disorder or inflammation, the microbial community is dominated by anaerobic bacteria and has the characteristics of high diversity. This could results in pH > 4.5. Simultaneously promote oxidation and nitrification reactions. The associated pro-inflammatory cytokines and ROS are increased, and metabolites in female reproductive tract are converted from lactic acid to biogenic amines. In addition, microbial co-infection can damage epithelial cells and disrupt the mucosal barrier.

The state of the microbiome is dynamic. The microbiome remains in dynamic balance when the body is healthy, whereas high bacterial diversity and low numbers of lactic acid bacteria indicate a bacterial imbalance or inflammation (Figure 1). Furthermore, many factors are reported to affect the balance of microbiome. On the one hand, epidemiological factors, such as dietary habits, contraception, smoking and sex life, are the contributing factors. On the other hand, social environment factors (sanitary conditions, living area, and socioeconomic) also influence the composition of the microbiome. Most notably, the host’s factors are also a kind of factor that cannot be ignored (Audirac-Chalifour et al., 2016; Torres-Poveda et al., 2016). *Lactobacillus* does not dominate all vaginal microbiome regions in healthy women, possibly due to geographical and socioeconomic influences. In Hispanic women and women of African descent (30–40%), a non-lactobacillus predominance of the vaginal microbiome is more common (Fettweis et al., 2014; Borgdorff et al., 2017; Laniewski et al., 2018). Meanwhile, in another study of Japanese-ancestry, European-ancestry, and African-ancestry, the vaginal microbiome, dominated by lactic acid bacteria, is more common in Japanese and Caucasians than women of African descent, confirming this phenomenon (Zhou et al., 2010). Interestingly, these areas also tend to have higher cancer prevalence rates than areas where *Lactobacillus* is the dominant species (Miller et al., 2018), which is the subject of future study. The result may suggest that *Lactobacillus* is a major defender of the microenvironment protecting the female reproductive tract.

The dynamic balance of the vaginal microbiota could also be affected by the host’s hormone or immune system. Due to the effect of estrogen, the vaginal microbiome becomes more volatile during menstruation, relatively stable and less diverse after menstruation (MacIntyre et al., 2015). After menopause, the lack of estrogen in women caused an increase in *anaerobic bacteria* in the vaginal flora and a decrease in *Lactobacillus*. The composition of the vaginal microbiota is affected by estrogen, which affects the release of pro-inflammatory cytokines, chemokines, and vaginal antimicrobial peptides (AMPs) (Torcia, 2019). Evidence suggests that when estrogen is elevated, large amounts of glycogen are detected in vaginal epithelial cells. Furthermore, estrogen helps the vaginal epithelium cells to mature and produce α-amylase. Glycogen is degraded to produce simple sugars such as maltotriose, maltotetraose, and α-dextrins. Consequently, *Lactobacillus*, which cannot break down glycogen, relies on glycogen products to grow, forming colonies in the vagina that produce substances such as lactic acid to inhibit the growth of other bacteria (Spear et al., 2014).

### The Function of *Lactobacillus* in the Female Reproductive Tract

*Lactobacillus* plays a role in protecting the body and maintaining function in the female reproductive tract or gastrointestinal tract. The composition of the vaginal microbiota may influence local immunity and be involved in the occurrence of cervical cancer and clearance of the HPV. The vaginal microbiota, dominated by various species of *Lactobacillus*, may play a protective role against opportunistic infections (Audirac-Chalifour et al., 2016). After utilizing those decomposition products of glycogen, *Lactobacillus* produce lactic acid that keeps the pH of the vaginal environment below 4.5, and acidizes the mucosal surface (Valenti et al., 2018). Lactic acid could reduce the cytotoxicity of natural killer cells, induce the secretion of anti-inflammatory cytokine interleukin-10 (IL-10), and reduce the production of pro-inflammatory cytokine IL-12 in dendritic cells (Sun et al., 2017). Notably, lactic acid is also produced by cancer cells and identified as a tumor marker (Ilhan et al., 2019). Therefore, the origin of lactic acid and its relationship with cervical cancer remains to be studied. In addition, bacteriocins with antimicrobial properties are also produced from *Lactobacillus*. Bacteriocins, a class of AMPs, are widely found in living organisms. It cannot only resist the invasion of pathogenic microorganisms through direct bactericidal action, but also mediate the acquired immune response and regulate the inflammatory immune response. Its cytotoxicity and cell lysis activity plays an important role in anti-tumor activity. Furthermore, a healthy vaginal microbiota is also associated with elevated defensins, and a previous study found a significant decrease in AMPs levels in patients with BV compared to healthy women (Al-Nasiry et al., 2020). Apart from vaginal AMPs, other substances can improve the vaginal defense system. IgA and IgG neutralize the effects of pathogenic microorganisms at the site of infection, preventing them from binding to the vaginal epithelium and ingesting nutrients. Mannose-binding lectin (MBL) may also protect against vaginal invasion and infection. These metabolites could resist the adhesion and growth of other bacteria in the vaginal epithelium. Furthermore, *Lactobacillus* compete with pathogens by acquiring nutrients and occupying territories, thus act as repellents and preventing pathogens from adhering to the vaginal epithelium (Maldonado-Barragan et al., 2016). Another study report that the complement system would be activated by *Lactobacillus* that sticks to the vaginal epithelium. The consequence could be triggering the body’s defenses. When the bacteria absorb nutrients and attach to the cell’s epithelium, the immune system is activated, further controlling microbial growth (Chase et al., 2015; Laura et al., 2016). As mentioned, *Lactobacillus* is a significant factor that maintains the health of the female reproductive tract, and the lack of *Lactobacillus* may promote the overflow of several anaerobic bacteria associated with sexual transmission. Cervical lesions could be caused by these anaerobic bacteria.

### The Particularity of Dysregulation of Vaginal Microbial Environment

Notably, numerous data point out that not all *Lactobacillus* can protect the health of the female reproductive tract and the stability of the vaginal microbial environment. Unlike other *Lactobacillus*, *L. iners* are often found in communities where non-*Lactobacillus* are predominant (Gajer et al., 2012). *L. crispatus* appears to be the most protective lactic acid bacterium for host health, but several evidence suggest that *L. iners* is associated with disease (Oh et al., 2015; Kyrgiou et al., 2017), and *L. iners* are also found in women with vaginal microbial environment disorders caused by HIV, HPV, and HSV-2 (Borgdorff et al., 2014; van de Wijgert et al., 2014). Previous studies have found that the possible reason for *L. iners* association with diseases is related to H_2_O_2_ (Mitra et al., 2015; Laura et al., 2016). H_2_O_2_ is considered to be produced by *L. crispatus* rather than *L. iners*. The vaginal microbiota with *L. crispatus* that is the dominant bacteria is the most stable. This microbiome rarely causes infections. *L. crispatus* produces both D-lactic acid and L-lactic acid, but *L. iners* produces only L-lactic acid (Amabebe and Anumba, 2018).

D-lactic acid is only a bacterial product, while L-lactic acid is a bacterial product also produced by vaginal epithelial cells and bacteria (Witkin et al., 2013). High concentrations of D-lactic acid inhibit chlamydia infection due to the pH-dependent effects of the vaginal microenvironment (Witkin et al., 2013). *Chlamydia* infection can promote the persistence of HPV infection, which will be discussed later. D-lactic acid also prevents upper reproductive tract infections by regulating the production of extracellular matrix metalloproteinase inducer (EMMPRIN) in vaginal epithelial cells induced by L-lactic acid and inhibiting the production of matrix metalloproteinase-8 (MMP-8) (Alvarez-Olmos et al., 2004). In addition, vaginal α-amylase is associated with levels of D-lactic acid and other vaginal epithelial AMPs, but not L-lactic acid. The importance of vaginal α-amylase was also demonstrated because vaginal α-amylase is highest in women without vulvovaginal disease and lowest in women with BV (Nasioudis et al., 2015).

## Inflammatory Response to Deregulation of Vaginal Microbial Environment

As the main defender of the cervicovaginal microenvironment, *Lactobacillus* maintains the dynamic balance of the entire cervix-vaginal microflora. As mentioned, while the number of *Lactobacillus* significantly decreased and different anaerobic bacteria gradually dominated, the dynamic balance will be disturbed, leading to the imbalance of cervical and vaginal microorganisms. Mechanistically, increased microbial diversity leads to the augmented production of related pro-inflammatory cytokines and chemokines, which amplify the inflammatory response and increase the number of immune cells recruited (Round and Mazmanian, 2009; Torcia, 2019; Wiik et al., 2019; Norenhag et al., 2020). This phenomenon promotes immune dysregulation in the female reproductive tract, thus providing a suitable site for tumor development (Figure 1; Schwabe and Jobin, 2013). The mixed microbial infection could promote the replication, transcription and modification of HPV, and increase the incidence of cervical cancer.

Additionally, microbial co-infection could increase inflammation and damage epithelial cells (So et al., 2020), which is one of the mechanisms of cervical intraepithelial neoplasia (CIN) (Wiik et al., 2019; Norenhag et al., 2020). Acute inflammation and persistent infection turn into chronic inflammation, which can cause cytotoxic effects on normal cells, damage DNA, and eventually develop into cancer cells, leading to cervical cancer (Balkwill and Mantovani, 2001; Fernandes et al., 2015). The expression of E6 and E7 promoted the inhibition of apoptosis despite the DNA damage in the cells, leading to chromatin abnormalities (Kyrgiou et al., 2017). The microbiota corresponding to the microbiota imbalance of the female reproductive tract and the increase of microbiota diversity is CST-IV, and the female reproductive tract microenvironment dominated by CST-IV is more susceptible than other microbiota. Active microorganisms in the CST-IV microbiome have become a potential risk factor for the occurrence of cervical cancer (Champer et al., 2018). It is noteworthy that microbial metabolites in the cervix and vagina could also be altered by the phenomenon of microbial disorders (Ilhan et al., 2019). For example, with the increase of anaerobic or microaerobic bacteria, the metabolites in the female reproductive tract will change from lactic acid to amines (Nelson et al., 2015; Srinivasan et al., 2015). Glycochenodeoxycholate (GCDC) is a metabolic product of a host-microbial metabolism, which could inhibit the growth of some anaerobic or microaerobic bacteria usually found in bacterial vaginitis and microbiological disorders (Fiorucci and Distrutti, 2015; Ridlon et al., 2016). However, GCDC can induce inflammation and toxicity, and cause carcinogenesis of the host epithelial cells, when the threshold of GCDC concentration is exceeded (Tatsugami et al., 2012). Ilhan et al. (2019) showed that the concentration of GCDC was elevated with the failure of *Lactobacillus* and the continuous presence of inflammatory reactions. All suggest that *Lactobacillus* is an indispensable guardian of the female reproductive tract.

The occurrence of BV and pelvic inflammation (PID) can be influenced by cervicitis (Klein et al., 2019). Cervicitis is usually caused by infection of non-symbiotic microorganisms that express certain antigens, leading to persistent inflammatory infection, as explained previously (Klein et al., 2020a). It is noteworthy that infections of the vaginal microbial environment may also indirectly affect the development of ovarian cancer by inducing local inflammation and immune regulation.

## The Driving Effects of Microbiome on Cervical Cancer

The microbiota maintains a symbiotic relationship with the host, and the dynamic balance of the microbiota is one of the prerequisites for the organism to maintain health. When the balance of the microbiota is disturbed, the change may harm the health of the host, alter the physiological changes of the individual, and lead to the development of diseases, such as cancer. Previous studies reported that microbiota plays an increasingly significant role in the occurrence and development of cancer (Bhatt et al., 2017; Rajagopala et al., 2017). For instance, *Helicobacter pylori* invasion of the gastrointestinal tract could induce chronic inflammation, lead to malignant transformation of B cells, and ultimately to gastric adenocarcinoma. Recently, researchers have linked the bacterium *H. pylori* to colorectal tumors (Witkowska and Smolewski, 2013; Shmuely et al., 2014; Ayse et al., 2015). Microbiome dysregulation is often involved in colorectal cancer, and *Streptococcus* is one of the key drivers. It can not only promote the occurrence of colorectal cancer (Yu et al., 2017), but also affect the recovery of prognosis of colorectal cancer (Mima et al., 2016). There are also significant differences in the composition of the breast cancer microbiome between women with breast cancer and healthy people (Urbaniak et al., 2016). Meanwhile, the researchers found a high abundance of oral symbionts in the lower respiratory tract of lung cancer patients (Tsay et al., 2018). As a rich area of microbes, the female reproductive tract is not surprising that microbe dysregulation exists.

Meanwhile, numerous reports have shown that microbial groups promote cancer by inducing inflammatory reactions in recent years. It has been demonstrated in mice with colorectal tumors that the microorganisms in mice induce the activation of tumor-associated myeloid cells, resulting in increased IL-23 and IL-17 secretion (Grivennikov et al., 2013). The development of endometrial cancer could be accelerated by PID (Yang et al., 2015). *Escherichia coli* would only cause cancers in the presence of an inflammatory response (Arthur et al., 2014). Although the primary pathogenesis is estrogen overproduction, there is evidence that gut microbes can influence estrogen production to some extent. This may indicate a mechanism where the microbiome in different parts of the body may have a circuit that affects the physiological function of other parts. Taken together, these suggest that the influence of the microbiome on the body’s immune system is a mechanism for inflammation-related cancer.

### The Imbalance of Cervicovaginal Microorganisms Is the Basic Factor That Induces Inflammation and Cervical Cancer

Cervicovaginal microbiological disorders have become a key factor in inflammation, HPV infection and cervical cancer. As mentioned earlier, *Lactobacillus* competes with pathogens for territory in the vaginal epithelium and inhibits the growth and development of pathogens by secreting lactic acid, bacteriocins and H_2_O_2_. An additional benefit of *Lactobacillus* is that it could also activate the complement system, trigger a local immune response, and further control the pathogen against the organism (Kovachev, 2020). However, in addition to the most beneficial *Lactobacillus*, other microbes, such as anaerobic bacteria, can regulate the body’s immune response when they lose balance. This can illustrate the complex relationship between the female genital tract and cervicovaginal microorganisms. The progression of cervical cancer is related to changes in the composition of the microorganism in the cervix vagina, especially the failure of *Lactobacillus* and the overgrowth of anaerobic bacteria (Mitra et al., 2015; Audirac-Chalifour et al., 2016). Microbial imbalance is often associated with the decreased abundance of *Lactobacillus* and the increased quantity of anaerobic bacteria (Mitra et al., 2015; Sodhani et al., 2017). This is a typical characteristic of CST-IV in the microbial spectrum. It is interesting to note that the characteristics of bacterial vaginitis are analogous to the trait of CST-IV (Kovachev, 2020). The conclusion could suggest that bacterial vaginitis and CST-IV are closely linked.

### Human Papillomavirus Is a Key Factor in the Development of Cervical Cancer

Human papillomavirus is known to be a pivotal factor in the development of cervical cancer. HPV causes various diseases, including cervical cancer and precancerous lesions (Li et al., 2020). HPV is divided into hr-HPV and lr-HPV. Most of the HPV entering the female reproductive tract is cleared by the host’s immune system (Audirac-Chalifour et al., 2016). Toll-like receptors (TLRs), natural killer cells and other mechanisms may be activated by the host to eliminate HPV (Stanley, 2012). A local innate immune response may mediate the initial host immunity to HPV infection, and acquired immunity mediated by antigen-presenting cells after HPV vaccination can also reduce the infection rate of HPV. However, when the host is subjected to persistent hr-HPV infection, it can develop cervical squamous intraepithelial lesions, and even cervical cancer (Astride et al., 2016). These cancer-related viruses may cause cancer directly by affecting the cellular structure, or indirectly contribute to cancer through immune escape or chronic inflammation (Curty et al., 2019). E6/E7 may disrupt the regulation of gene expression in host cells, leading to abnormal cell proliferation by influencing the control mechanisms of cell cycle and antigen expression, leaving the body unresponsive (Wang et al., 2014; Zhang et al., 2015). In addition, the expression of E6/E7 also overexpressed PD-1, thereby inhibiting the activation of T cells (Allouch et al., 2020). The microenvironment of the female reproductive tract changes with the stage of cervical cancer when a woman has cervical cancer. Vaginal acidity and cytokines are also affected by the development of cervical cancer, leading to further local immunosuppression. For instance, the presence of immunosuppressant agents, such as transforming growth factor-β1 (TGF-β1) and IL-10, may contribute to HPV persistence. The cervicovaginal microflora can increase the expression of immunosuppressive factors (Kyrgiou et al., 2017). A previous study examines that serum FMS-like tyrosine kinase and tumor necrosis factor-α (TNF-α) expression are significantly elevated in patients with CST-IV disease compared to the CST-I group (Anahtar et al., 2015).

Human papillomavirus is a major contributor to cervical cancer and its precancerous lesions; however, HPV is not a sufficient condition for cervical cancer (Muñoz et al., 2003). The persistence of HPV infection can be affected in many ways. Imbalanced cervicovaginal microbiota and inflammation contribute to HPV persistence. Various cytokines are produced and damaged by the epithelial intimal barrier that creates optimal conditions for HPV infection (Astride et al., 2016). Persistent infection with HPV in turn impact the dysregulation and inflammation of cervical and vaginal microbiology, because the host’s immune defenses could be negatively affected by persistent HPV infection (Klein et al., 2019). A previous study has found that HPV may also affect the mucosal metabolism, and further affect the cervix vaginal microenvironment. When HPV infects the mucosal surface, a series of inflammation-related mechanisms are initiated by HPV, such as the activation of overexpressed macrophages and NK cells, and the activation of local mucosal immunity by pro-inflammatory cytokines (Garcea et al., 2005). HPV infection, bacterial vaginitis and microbiota dysregulation are capable of acting on or influencing each other mutually.

### Inflammation Is an Important Driver of Human Papillomavirus Infection and Cervical Cancer

Chronic or persistent infection with HPV is necessary, but HPV alone is not enough to induce cervical cancer and require additional endogenous or exogenous cues. At the low abundance of Lactobacillus, there is an increased likelihood of dysregulating the cervix vaginal microbiota and an increased risk of bacterial vaginitis, leading to increased production of mucin-degrading enzymes. Meanwhile, the production of H_2_O_2_, lactic acid and other products are reduced because of fewer producers, and the barrier of cervical and vaginal epithelium mucosa becomes more fragile (So et al., 2020). The microflora stimulates the production of pro-inflammatory cytokines that further disrupt the epithelial intimal barrier. A previous survey reported 32 women (ages 38–55) diagnosed with cervical cancer (Kovachev, 2020), to determine whether or not they had suffered from bacterial vaginitis and observed their cervix vaginal microflora. The results showed that 23 (71.9%) patients are affected by a vaginal microbiome disorder. Another study supports the hypothesis that inflammation could contribute to cervical cancer. Elevated inflammatory cytokines are found in the vaginas of patients with cervical cancer or precancerous lesions (Mhatre et al., 2012; Caselli et al., 2020). In 85 patients (32%), the prevalence of CST-IV is significantly higher than the general population (10.3%). Meanwhile, compared with patients with CIN2, CST-IV is more common in patients with CIN3 stage. These data illustrate the correlation between the severity of CST-IV and cervical cancer. The study also noted that high concentrations of pro-inflammatory cytokines are found in the vaginal environment of CIN patients. Compared with healthy people, IL-1α, IL-1β, IL-6, IL-8, and TNF-α in the vagina of CIN patients are higher. Interestingly, the CST-IV ratio is also higher in these patients with the cervicovaginal microbial spectrum (Di Paola et al., 2017; Gosmann et al., 2017). In Mitra’s study, by examining the microbiota of 169 women, they found that the rate of a CST IV vaginal microbiome doubled in women with low-grade squamous intraepithelial lesions (LSIL), tripled in women with highly squamous intraepithelial lesions (HSIL), and quadrupled in women with invasive cancer (Mitra et al., 2015).

Multiple cytokines are involved in developing various tumors, starting with high-risk HPV infected cells, through maladjustment of many pathways (Barbisan et al., 2012). In the innate immune system, TLRs are used to activate inflammatory defense mechanisms and regulate the clearance of immune complexes, but can also lead to chronic inflammation of unhealthy tissues. Therefore, imbalance or disturbance in one part can promote the formation of tumors (Jouhi et al., 2014). Mutated TNF-α can directly lead to cell canceration (Yang et al., 2018). Meanwhile, IL-6, IL-8, IL-1β, and other inflammatory cytokines can promote tumor proliferation and the development of cervical cancer (Schäfer et al., 2013; Liu et al., 2020). For example, IL-1β regulates the expression of CCL-2 by activating nuclear factor-κ-gene binding (NF-κB). It was also found that TGF-β mediated immune escape by promoting the overexpression of FBP1 gene in NK cells, making NK cells in a state of low activity in tumor microenvironment (Cong et al., 2018).

Abnormal activation of several different signaling pathways is also a factor contributing to the occurrence of cervical cancer. The upstream regulatory regions of some transcription factors, such as NF-κB and signal transducers and activators of transcription 3 (STAT3), contain homeopathic elements associated with carcinogenesis (Gupta et al., 2018). These transcription factors may influence the regulation of the HPV genome (Shukla et al., 2019). Activation of the janus kinase (JAK)/STAT pathway plays an important role in immune escape. STAT3 can not only increase the expression of inhibitory cytokines such as TGF-β, IL-6, and IL-10, but also promote the aggregation of regulatory T cells and induce the immunosuppressive microenvironment by inhibiting the maturation of dendritic cells (Yu et al., 2007). E6/E7 oncoprotein expression has been found to promote the expression of STAT3, which in turn inhibits the production of PRb and p53 (Arany et al., 2002; Basukala et al., 2019). In addition, STAT5 is also activated by oncoprotein E7. Interestingly, this is also required for HPV31 genome amplification (Hong and Laimins, 2013). NF-κB plays an essential role in immune response, inflammatory effects, viral replication and tumorigenesis (Bonab et al., 2021). Mutations in HPV E6/E7 oncoprotein IκB down-regulate NF-κB, thereby blocking the immune response. After developing cancer, NF-κB is reactivated by cytokines released by M2 macrophages. Dysregulation of NF-κB promotes inflammatory responses, abnormal cell proliferation and differentiation, persistent angiogenesis, avoidance of immune destruction, and even tissue infiltration and metastasis. Interestingly, while E6 induces IL-6 expression through NF-κB, IL-6 also promotes STAT3 production and activation. This also demonstrates the phenomenon of pathways in series with each other (Pique et al., 2019).

Of note, oxidative and nitrifying stress is also one of the main mechanisms of inflammation-induced cancer. Some oxygen and nitrogen-related compounds have been linked to inflammation-related cancer caused by microbes (Kipanyula et al., 2013; Nelson et al., 2015). They can cause abnormal oxidative stress in the microenvironment and inhibit the function of immune cells. A previous study has found that unlike CST with *Lactobacillus* as the dominant species, CST-IV with anaerobes as the dominant species has a higher concentration of amines (Nelson et al., 2015). These anaerobic bacteria can increase the level of biogenic amine production. Biogenic amines not only lead to nitrosamine production but also improve the resistance of pathogens to host-mediated defense systems. Additionally, biogenic amines have been found to promote biofilm formation in some pathogens. Certain species of *Lactobacillus* prevent the colonization of bacteria that produce high levels of amines. *Lactobacillus* could also remove the carcinogens of these amines and provide an extra layer of protection. This is because *Lactobacillus* is the dominant species in the cervicovaginal microbiological community type of amines less reason. In addition, *Lactobacillus* has a cytotoxic effect on cervical cancer cells, preventing the development of cervical cancer (Kyrgiou et al., 2017). Interestingly, inhibition of glucose metabolism could significantly reduce the clearance capacity of helper T cells (Choi et al., 2018). Cancer cells can also drive metabolic changes that suppress immune responses (Bader et al., 2020). These suggest that immune response is related to metabolic pathways, and immune metabolism may be a new way to treat tumors.

**FIGURE 2:**
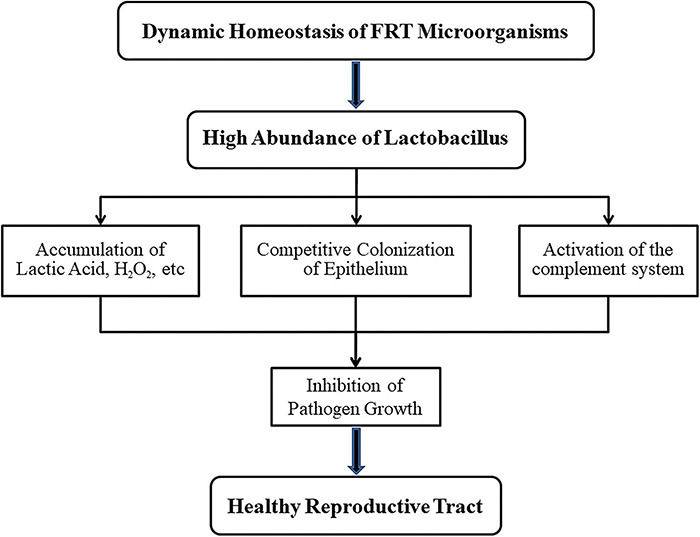
Mechanisms of *lactobacillus* maintaining health for female reproductive tract (FRT). When the microbial community of female reproductive tract is in dynamic balance, *lactobacillus* with high abundance will not only produce lactic acid, H_2_O_2_ and other products but also compete with anaerobic bacteria for vaginal epithelial cells. In addition, the complement system could be activated by *lactobacillus*. These features prevent the growth of pathogens and thus ensure the health of the host.

**FIGURE 3:**
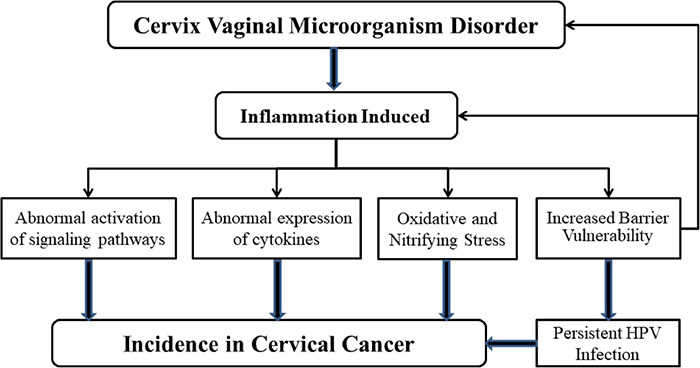
Mechanisms of microbial dysregulation and inflammation leading to cervical cancer. When the balance of microbial environment of female reproductive tract is upset, the inflammatory response is activated. H_2_O_2_, the amount of lactic acid decreases because of the increase in the number of anaerobic bacteria. At the same time, the production of a large number of pro-inflammatory cytokines affects the protective ability of the mucosal epithelial barrier. These phenomena can increase the likelihood of persistent HPV infection, which can lead to cervical cancer. Abnormal expression of cytokines and abnormal activation of signal pathways also lead to the occurrence of cervical cancer. It is important to note that the presence of HPV could also influence microbial homeostasis and the activation of inflammation-related mechanisms.

Human papillomavirus infection, inflammation and microbiome dysregulation interact or influence each other. We respectively summarized the mechanism of changes in the reproductive tract microenvironment under the conditions of *Lactobacillus*-dominated vaginal microbial environment and cervical and vaginal microbiota disorders, as shown in Figures 2, 3.

### Carcinogenic Effects of Microorganisms

An increase in certain bacterial or microbial diversity may serve as a biomarker for cervical changes to identify the risk of persistent hr-HPV infection, CIN, and even cervical cancer (Norenhag et al., 2020). Recently, the CST-IV microflora spectrum has become a potential risk factor for cervical cancer (Torcia, 2019). One of the dominant anaerobes is carcinogenic, such as *Gardnerella*, *Sneathia*, *Fusobacterium* spp., and *Atopobium vaginae* (Drago et al., 2016; Brotman et al., 2018). High levels of *Gardnerella*, *A. vaginae*, and *Sneathia* spp. were most common among women who are persistently infected with hr-HPV for 1 year. Moreover, these bacteria are the common anaerobic bacteria in BV. Interestingly, in addition to these anaerobic bacteria, *L. iners* is also abundant (Di Paola et al., 2017). Large amounts of *A. vaginae* are inversely associated with HPV (Brotman et al., 2018). In addition, some studies have found that high concentrations of *Sneathia* spp. and *Fusobacterium* spp. are found in the genital tract environment of patients with the squamous intraepithelial disease and cervical cancer. In the meantime, *mycoplasma* and *chlamydia* have also entered the research field, and increasing numbers of reports have found a link between them and persistent HPV infection.

#### *Fusobacterium* spp.

*Fusobacterium* spp. has been discovered in colorectal cancer. The proinflammatory expression patterns are reported in colorectal tumors in the presence of high *Fusobacterium* spp. abundance (Kostic et al., 2013). This may indicate that microbe-induced inflammation is a mechanism that leads to cancer. Furthermore, *Fusobacterium* spp. could promote the development of dysplasia (Norenhag et al., 2020). This showed that a strong link between gut microbiome populations and colorectal cancer (Viaud et al., 2013). It is possible to guess that the cervix vaginal microbiota may be linked to cervical cancer in the same manner. It has been reported that *Fusobacterium* spp. is often found in the female reproductive tract (Sommer et al., 2017). In the Korean twin cohort, *Fusobacterium* spp. serves as a microbial marker to observed HPV infection. In addition, the cytokine profile showed that the local levels of interleukin-4 (IL-4) and TGF-β1 were significantly increased in the cervix vaginal microflora dominated by *Fusobacterium* spp. in a *Lactobacillus* deficiency, and high mRNA levels of TGF-β1, IL-4, and IL-10, which was also found in cervical biopsies from patients with the squamous intraepithelial disease and cervical cancer (Audirac-Chalifour et al., 2016). Another study demonstrates that IL-4 levels are elevated in the presence of *Fusobacterium* spp. infection (Vielot et al., 2015). *Fusobacterium* may be involved in an immunosuppressive microenvironment characterized by anti-inflammatory cytokines. This indicates that *Fusobacterium* spp. plays a certain role in the occurrence of cervical cancer, and further research is needed to determine whether there is a new influencing mechanism.

#### Mycoplasma genitalium

The *Mycoplasma* family consists of the genera *Mycoplasma urealyticum* and several relatively common sexually transmitted species, such as *Mycoplasma hominis* and *Mycoplasma genitalium*. They are associated with cervical inflammation (Astride et al., 2016; Mitra et al., 2016; Klein et al., 2020b).

The common mycoplasmas that infect the female genital tract are *M. genitalium* and *M. hominis*. Interestingly, they are commonly found as non-symbiotic bacteria in patients with cervicitis and bacterial vaginitis (Klein et al., 2019). Klein’s team found that *M. genitalium* can infect epithelial cells, causing a series of intracellular infections and breaking tight junctions, leading to bacterial vaginitis and cervicitis, and increases the incidence of cervical lesions (Klein et al., 2019). In addition, some studies have confirmed that *M. genitalium* is an independent pathogenic microorganism leading to cervicitis (Taylorrobinson and Jensen, 2011; Ye et al., 2018). This may indicate that in the case of HPV infection, *Mycoplasma* can cause infect both the cell inside and outside. Other studies have shown that infection of *M. genitalium* induces chromosomal lesions in cells, which may lead to cancerous cells, especially under chronic inflammation (Verteramo et al., 2009).

The relationship between *Mycoplasma* and cervical lesions has been gradually discovered, and some scholars have conducted epidemiological studies on them. However, the mechanism between *Mycoplasma* and cervical cancer is rarely explored. Further studies on the molecular mechanism are needed in the future.

#### Chlamydia trachomatis

*Chlamydia trachomatis (CT)* is the most prevalent sexually transmitted infection (STI) worldwide. Lv et al. (2019) reported that CT-positive women had an approximately four-fold higher risk of hr-HPV infection than those who were *CT*-negative. This suggests that *CT* may increase the susceptibility of the cervical gland to HPV. Besides, other studies have found that *CT* infection can prolong the persistence of HR-HPV infection to the cervix (Verteramo et al., 2009; Silva et al., 2014; Vielot et al., 2015). This further supports the association between *CT* and HPV. Escarcega-Tame et al. (2020) also found that among women with high levels of CIN, the prevalence of *CT* was 47 percent higher than in healthy women.

*Chlamydia trachomatis* infection can damage the cervical mucosal barrier and allow hr-HPV to infect the cervical epithelium through its own entrance (Erika et al., 2005; Paavonen, 2012). Meanwhile, *CT* could also induce chronic inflammation and affect local and cellular immunity of the cervix. This inhibits the ability of an organism to clear HPV. In addition, several studies have documented that *CT* infection leads to overexpression of the E6/E7 oncogene of HPV (Paavonen, 2012). Interestingly, cervical lesions could be promoted by *CT* and HPV through co-infection. *CT* and HPV may alter cell-to-cell adhesion and differentiation by inducing the expression of pro-inflammatory mediators. Silva et al. (2014) found that *CT*-infected cervical cancer cell lines were found to secrete more pro-inflammatory cytokines. At the same time, *CT* may enhance HPV activity (Fischer, 2002).

There is a lack of studies on the effect of non-bacterial components (*Chlamydia*, *Mycoplasma*, *fungal*, and non-HPV viruses) on cervical cancer, which may be a direction worth exploring. In recent years, some reports have also found that they may affect the progression of cervical cancer (Liang et al., 2019; Stelzle et al., 2021; Subramaniam et al., 2021).

#### Gut Microbiomes

The intestinal microbiome can indirectly affect the abundance of *Lactobacillus* in the cervix vaginal microenvironment by regulating the release of estrogen (Romero et al., 2014; Jespers et al., 2017). This may lead to a new concept, the gut-vaginal axis (Baker et al., 2017). Some microorganisms in the human intestine can affect the secretion and transport of estrogen. They secrete β-glucuronidase and β-glucosidase. These products bind to estrogen in the liver and promote its reabsorption into the circulation (Plottel and Blaser, 2011; Flores et al., 2012). Free estrogen is sent to the distal sites, including the female reproductive tract. It binds to receptors and triggers a cascade of intracellular signaling that triggers glycogen production and thickens the genital epithelium and produces mucus. Before menopause, estrogen levels drop dramatically before menstruation and the *Lactobacillus* in the vaginal microbiome collapse (Mirmonsef et al., 2016; Muhleisen and Herbst-Kralovetz, 2016; Nunn and Forney, 2016). When *Lactobacillus* concentrations are too low, anaerobic bacteria become the dominant bacteria in the vaginal microenvironment contributing to the development of cervical cancer. Gut microbes regulate the enterohepatic circulation of estrogen. Their circulatory effects can be felt throughout the body, such as the cervix and the mammary glands (Goedert et al., 2015; van der Meulen et al., 2016). A previous study has found that cervical cancer patients have a unique gut microbiome (Wang et al., 2019). In addition, a lack of estrogen may also cause vaginal atrophy. Both are thought to be responsible for increasing bacterial diversity (Brotman et al., 2018).

#### Other Microbiome

*Gardnerella vaginalis* may be involved in biofilm formation, contributing to the persistence of HPV infection (Swidsinski et al., 2014; António and Nuno, 2015). Berggrund et al. (2020) studied the relationship between the cervix vaginal microflora and the course of HPV-16 infection. They found that *G. vaginalis* and *A. vaginae* were associated with CIN (Curty et al., 2017; Di Paola et al., 2017; Godoy-Vitorino et al., 2018; Berggrund et al., 2020). A high abundance of *Atopobium* spp., in the cervix vaginal microflora, may be one of the critical markers of cervical lesions (Seo et al., 2016). *Atopobium* spp. is highly specific for BV. Similar to *Gardnerella*, the enrichment of *Atopobium* spp. is also related to CIN3 to some extent (Brusselaers et al., 2019). *A. vaginae* can activate pro-inflammatory transcription factor-κB (NF-κB), TNF-α, IL-6, and IL-8 (Doerflinger et al., 2014). In addition, patients with squamous intraepithelial disease tend to have high levels of *Sneathia* spp. in the female reproductive tract (Audirac-Chalifour et al., 2016).

## Treatment of Cervical Cancer

Surgery, chemotherapy and radiotherapy can cure more than 90% of women with early cervical cancer; however, there are still cancer recurrences or metastasis. Many efforts have been made to develop new drugs to treat cervical cancer.

### Probiotic Therapy

Probiotics are made up of living bacteria such as *Bifidobacterium*, *Lactobacillus*, and *Streptococcus*. It reduces the risk of certain cancers by regulating the composition of the microbiome and increasing inflammation (Li et al., 2020).

Probiotics containing *Lacticaseibacillus Paracasei Shirota* or *Lacticaseibacillus casei* can significantly improve HPV clearance (Verhoeven et al., 2013). In addition, *Bacteroides fragilis* can also improve the effectiveness of CTLA-4 targeted immunotherapy. The combination of *L. gasseri* and *L. crispatus* has shown *in vitro* cytotoxic effects on HeLa cervical cancer cells (Motevaseli et al., 2013a, b). Interestingly, milk consumption containing *Lacticaseibacillus rhamnosus* and *Bifidobacterium lactate* increased NK cell activity, and some *Lactobacillus* also stimulated the production of cytokines such as TNF-α and γ-IFN, which in turn increased immune cell activity (Bermúdez-Humarán et al., 2011; Dallal et al., 2012; Shin et al., 2016; Pique et al., 2019). Intranasal administration of recombinant *Lactococcus lactis* expressing E7 antigen can also induce the body to produce antibodies to E7, thus initiating specific immunity (Bermúdez-Humarán et al., 2011; Carse et al., 2021). We summarized the probiotic, *in vivo* and *vitro* development and mechanism in Table 1, providing a reference for readers.

**TABLE 1:** Therapeutic potential of probiotics based on microbiome and inflammatory in cervical cancer.

	**Mechanism**	***In vitro/vivo***	**References**
*Lacticaseibacillus Paracasei Shirota*	Improving HPV clearance	*In vivo*	Verhoeven et al., 2013
*Lacticaseibacillus casei*	Improving HPV clearance	*In vivo*	Verhoeven et al., 2013
*Bacteroides fragilis*	Improving the effectiveness of CTLA-4 targeted immunotherapy	*In vivo*	Motevaseli et al., 2013a
*Lactobacillus gasseri*	Presenting cytotoxic effect in HeLa cell	*In vitro*	Motevaseli et al., 2013b
*Lactobacillus crispatus*	Presenting cytotoxic effect in HeLa cell	*In vitro*	Motevaseli et al., 2013b
*Lacticaseibacillus rhamnosus*	Increasing NK cell activity	*In vivo*	Bermúdez-Humarán et al., 2011
*Bifidobacterium lactate*	Increasing NK cell activity	*In vivo*	Dallal et al., 2012
*Lactococcus lactis*	Producing E7 antigen	*In vivo*	Bermúdez-Humarán et al., 2011

### Immunotherapy

Immunity is a kind of self-protection system of the body, which plays an important role in protecting the body against infections. The importance of immune checkpoints is highlighted. PD-1 receptor, the mediator of immune checkpoint, also plays a vital role in developing tumor. Typically, the PD-1 pathway regulates specific immunity, which in turn prevents cells from becoming cancerous (Allouch et al., 2020). When the PD-1/PD-L1 pathway is activated, T cell receptor signal transduction is inhibited and T cell activity is significantly weakened. Therefore, PD-1 mab drugs have been used clinically to specifically block the binding between PD-L1 and PD-1, reactivate the activity of T cells, and then kill tumor cells (Riley, 2009). The use of PD-1 mab drugs has also been approved by the FDA and is already being used to treat advanced cervical cancer. In addition, some STAT3 or NF-κB inhibitors are in pre-clinical development (Pakish and Jazaeri, 2017). However, due to high toxicity, the development of these inhibitors in tumor therapy has been slow. Therefore, many studies have focused on regulating upstream pathway targets to inhibit STAT3 or NF-κB activation. At present, there are clinical treatment options for IL-6 expression (Johnson et al., 2018). Proteasome inhibitors that block IκB have also been found to reduce NF-κB phosphorylation and treat multiple myeloma (Vrabel et al., 2019).

## Conclusion and Future Perspectives

Microorganisms maintain a symbiotic relationship with the host. The host provides a suitable environment for the microorganism, and they can regulate the body function of the host to a certain extent. Opportunistic pathogens, or pathogens, trigger a series of reactions that can infect the body and even lead to cancer when the balance between the microbe and the host is disrupted. Additionally, with the deepening of research, an increasing number of studies have found that microorganisms can cause local inflammatory reactions, thus increasing the susceptibility of the body and the risk of cancer. In the female reproductive tract, the role of Lactobacillus has become prominent, and when the dynamic balance of the microbial environment is upset, the negative effects of anaerobic bacteria will counteract the positive effects of Lactobacillus. How to maintain the dynamic balance is something to consider.

Microbial dysregulation also results in the release of a large number of pro-inflammatory cytokines and chemokines, resulting in a local inflammatory response. The link between vaginal microbes and cancer has also been widely investigated. Some studies have also shown that lactic acid bacteria have cytotoxic effects on cervical cancer cells. The effects of *Mycoplasma* and *Chlamydia* on the body should also be explored. Some of it may be in the body itself, but there is also the possibility that conditions can cause disease. If it is an exogenous substance, it is more important to consider whether the immune system of the body can remove it.

The effect of intestinal microbes on cervical cancer is worth discussing. In cervical cancer, gut microbes have been found to regulate the release of estrogen. However, the role of intestinal microbes in tumor regulation is more elaborate. The tumor microenvironment is simultaneously regulated by immune cells, related pathways, and chemokines, and gut microbes have been found to influence the expression of inflammation-related pathways. When intestinal microbes are imbalanced, it also inhibits immune monitoring to a certain extent. The type of gut microbiome can block the PD-1/PD-L1 pathway, and thus the composition of gut microbiome can determine the clinical response of patients to immune checkpoint inhibitors (Vétizou et al., 2015; Routy et al., 2018; Tanoue et al., 2019). Interestingly, intestinal microbes can also play a certain regulatory role in other cancers, even neurological diseases, so it is also worth discovering whether microbes from other parts of the body, such as breast and lung, can significantly impact cervical cancer. In conclusion, microorganisms are potential targets for mediating tumor therapeutic efficacy. The use of probiotics, customized oral microbial cocktail tablets, may be an effective option and development direction for treating cervical cancer, and even other cancers.

Human papillomavirus is believed to be a critical driving factor in the development of cervical cancer. The infection process and signaling pathway of HPV have been well studied. These studies provide different ideas and directions for the treatment of cervical cancer. It is also essential to identify reliable biomarkers for the diagnosis of cervical cancer. NF-κB is known to mediate the physiological function of the immune response and the innate immunity after infection. Inhibition of NF-κB may also inhibit the anti-tumor effect of the immune system balance. Therefore, the balance between the two needs to be considered. Different members of the STAT family may also combine to form heterodimers, which may also play an important role in the immune system of the body. It is worth studying how the balance of STAT family members influences tumor progression or immunity.

Inflammation is not always caused by infection, but infection and other foreign substances entering the body often cause inflammation. However, inflammation is a quick fix, not a final solution. Clearing the cause still requires precise, antigen-specific adaptive immunity compared to innate immunity. Unfortunately, immune recognition is based on specific antigens. With the interference of foreign substances or some abnormal expression of cytokines, the immune system may not recognize all the different cells in the primary site. This is also why HPV or cervical cancer cells achieve immune escape. All direct therapies against cancer, including surgery, radiotherapy and chemotherapy, must involve immunity for achieving sustained efficacy. Inflammation is the leading cause of cancer, and the mechanism between immunity and cancer deserves further study.

Although the biology of various tumors differs, there are some common causes of clinical symptoms. In these cancers, such as gastric adenocarcinoma, colon cancer, and rectal cancer, imbalances of microorganisms are believed to cause chronic inflammation. Ineffective immune responses that fail to clear infections can lead to immune tolerance, which fosters cancer growth. The reality today is immunotherapy is often used as a last resort. We believe that ensuring that specific immunity is not suppressed is the basis of immunotherapy and should be used throughout the whole process.

As compiled above, we should focus on microorganisms and inflammation as two crucial factors in detecting cervical lesions, which may effectively treat cervical cancer.

## Author Contributions

Z-WZ contributed to the conception and overall idea of the study and completed the manuscript. YC, H-ZL, D-DW, and H-YL helped in searching for related articles. H-ZL, YC, H-YL, D-DW, and L-CG revised the manuscript. All authors contributed to the article and finally the submitted version is approved by L-CG.

## Conflict of Interest

The authors declare that the research was conducted in the absence of any commercial or financial relationships that could be construed as a potential conflict of interest.

## Publisher’s Note

All claims expressed in this article are solely those of the authors and do not necessarily represent those of their affiliated organizations, or those of the publisher, the editors and the reviewers. Any product that may be evaluated in this article, or claim that may be made by its manufacturer, is not guaranteed or endorsed by the publisher.

## Abbreviations

CSTs: community state types
CT: *Chlamydia trachomatis*
HPV: human papillomavirus
BV: bacterial vaginosis
CIN: cervical intraepithelial neoplasia
AMPs: antimicrobial peptides
MBL: Mannose-binding lectin
GCDC: glycochenodeoxycholate
PID: pelvic inflammation
LSIL: low-grade squamous intraepithelial lesions
HSIL: highly squamous intraepithelial lesions
CVL: cervix vaginal lavage
STI: sexually transmitted infection
ROS: reactive oxygen species
KG: Kaempferol-7-O-b-D-glucoside
EMMPRIN: extracellular matrix metalloproteinase inducer
MMP-8: matrix metalloproteinase-8
MMP: matrix metalloproteinase
IL: interleukin
TGF- β 1: transforming growth factor- β 1
TNF- α: tumor necrosis factor- α
TLRs: toll-like receptors
STAT: signal transducers and activators of transcription
JAK: janus kinase
NF- κ B: nuclear factor- κ-gene binding.
